# Loss of AND-34/BCAR3 expression in mice results in rupture of the adult lens

**Published:** 2009-04-03

**Authors:** Richard I. Near, Richard S. Smith, Paul A. Toselli, Thomas F. Freddo, Alexander B. Bloom, Pierre Vanden Borre, David C. Seldin, Adam Lerner

**Affiliations:** 1 Department of Medicine, Section of Hematology and Oncology, Boston Medical Center, Boston, MA; 2 Department of Pathology, Boston University School of Medicine, Boston, MA; 3 The Jackson Laboratories, Bar Harbor, ME; 4 Department of Biochemistry, Boston University School of Medicine, Boston, MA; 5 School of Optometry, University of Waterloo, Waterloo, Ontario

## Abstract

**Purpose:**

AND-34/BCAR3 (Breast Cancer Anti-Estrogen Resistance 3) associates with the focal adhesion adaptor protein, p130CAS/BCAR1. Expression of AND-34 regulates epithelial cell growth pattern, motility, and growth factor dependence. We sought to establish the effects of the loss of AND-34 expression in a mammalian organism.

**Methods:**

AND-34**^−/−^** mice were generated by homologous recombination. Histopathology, in situ hybridization, and western blotting were performed on murine tissues.

**Results:**

Western analyses confirmed total loss of expression in AND-34**^−/−^** splenic lymphocytes. Mice lacking AND-34 are fertile and have normal longevity. While AND-34 is widely expressed in wild type mice, histologic analysis of multiple organs in AND-34**^−/−^** mice is unremarkable and analyses of lymphocyte development show no overt changes. A small percentage of AND-34**^−/−^** mice show distinctive small white eye lesions resulting from the migration of ruptured cortical lens tissue into the anterior chamber. Following initial vacuolization and liquefaction of the lens cortex first observed at postnatal day three, posterior lens rupture occurs in all AND-34**^−/−^** mice, beginning as early as three weeks and seen in all mice at three months. Western blot analysis and in situ hybridization confirmed the presence of *AND-34* RNA and protein in lens epithelial cells, particularly at the lens equator. Prior data link AND-34 expression to the activation of Akt signaling. While Akt Ser 473 phosphorylation was readily detectable in AND-34^+/+^ lens epithelial cells, it was markedly reduced in the AND-34**^−/−^** lens epithelium. Basal levels of p130Cas phosphorylation were higher in AND-34^+/+^ than in AND-34**^−/−^** lens epithelium.

**Conclusions:**

These results demonstrate the loss of AND-34 dysregulates focal adhesion complex signaling in lens epithelial cells and suggest that AND-34-mediated signaling is required for maintenance of the structural integrity of the adult ocular lens.

## Introduction

*AND-34* was originally identified as a gene whose expression was upregulated in the thymus of “AND” T cell receptor transgenic mice following the induction of thymocyte apoptosis through cross-linking of the T cell receptor [1]. *AND-34* was found to encode a 95 kDa protein that is bound by its carboxyl terminus to the focal adhesion adaptor proteins, p130Cas (Crk-associated substrate) and HEF1 (human enhancer of filamentation) [2,3,4]. Interest in this protein has focused on the ability of BCAR3 (Breast Cancer Anti-Estrogen Resistance 3), the human homolog of AND-34, to induce anti-estrogen resistance in breast cancer in concert with p130Cas (BCAR1) [5-8]. Expression of the BCAR3/p130Cas complex is generally higher in mesenchymal than epithelial breast cancer cell lines [9]. Overexpression of AND-34/BCAR3 in epithelial cells induces high levels of extracellular fibronectin deposition as well as increased migration and colocalization with p130CAS at the cell membrane [9,10]. Conversely, depletion of AND-34/BCAR3 in mesenchymal breast cancer cells by RNA interference inhibits cell migration and invasiveness and relocalizes p130CAS away from the membrane [10]. These studies have served to highlight the role of AND-34/BCAR3 and p130Cas in cell adhesion and migration signaling pathways.

AND-34/BCAR3 is a member of the NSP (novel SH2-containing protein) family of proteins [11]. While NSP1 is expressed in humans but not mice, NSP2 (AND-34/BCAR3) and NSP3 (CHAT [Cas/HEF1-associated signal transducer]/SHEP [SH2 domain-containing Eph receptor-binding protein 1]) are expressed in both species [12,13]. All NSP proteins contain an NH_2_-terminal SH2 (Src homology domain 2) domain, a central proline/serine-rich domain, and a COOH-terminal domain with modest homology to Ras subfamily GDP-exchange factors (GEFs). In each case, there is association with p130CAS [3,12,14] or the related p130CAS family members HEF1 or Sin (Src interacting) [4,15]. Just how much functional redundancy exists among the NSP homologs has not been well studied, although various splice variants have been reported in the same breast cancer cell lines [16]. However, one study recently identified AND-34/BCAR3 but very little NSP3 or NSP1 protein in human breast cancer lines [9].

As NSP family members contain a GEF-like domain, several studies have examined whether NSP proteins activate Ras subfamily GTPases. While initial transfection studies using chimeric GST (glutathione-S-transferase)-GTPase assays suggested that AND-34 could activate Ral, Rap1, and R-Ras [3] and recent structural studies confirm that the AND-34 COOH-terminus has a Cdc25-like GEF fold [17], subsequent studies using pull-down assays have not detected robust activation of these GTPases [4,18,19]. In those studies that did identify activation of Rap1 by the murine NSP3 member CHAT, activation was likely indirect through activation of the p130Cas-associated Rap1 GEF C3G [20]. In contrast to the generally negative results with Ras subfamily GTPases, overexpression of AND-34 in epithelial or lymphoid cell lines reproducibly activates the Rho subfamily GTPases, Rac and Cdc42 [4,7]. Not surprisingly, given the absence of a Dbl-like Rho subfamily GEF domain, the ability of AND-34 to activate Cdc42 and Rac is indirect and apparently dependent on PI3K (phosphatidylinositol-3-kinase) activation [19]. Such PI3K dependent signaling in AND-34 overexpressing cells also induces the activation of Akt kinase as judged by serine 473 phosphorylation [19].

Among the three NSP family members, only AND-34/BCAR3 induces anti-estrogen resistance in estrogen receptor (ERα)-positive breast cancer cell lines [9]. Surprisingly, although Rac activation has been implicated in anti-estrogen resistance in breast cancer cells, activation of Rac or Cdc42 by AND-34 is insufficient to induce anti-estrogen resistance as overexpression of any of the three NSP family members activates these GTPases [9]. More recent comparison studies demonstrated that among the three NSP family members, AND-34 induces the most robust serine phosphorylation of p130Cas (unpublished communication). The physiologic consequences of AND-34-mediated p130Cas serine phosphorylation remain a subject of active investigation.

Given the possibility that disruption of the AND-34/p130Cas complex might be therapeutically beneficial in anti-estrogen refractory breast cancer, we sought to establish the normal physiologic role of AND-34 by using homologous recombination to eliminate expression of AND-34 in mice. Here, we report that while AND-34**^−/−^** mice are healthy and fertile, they display an unusual ocular lens phenotype. In an age-dependent fashion and with very high penetrance, AND-34**^−/−^** mice undergo rupture of the lens capsule with occasional migration of a portion of the extruded lens into the anterior or posterior chamber of the eye. Imaging and biochemical studies demonstrate that AND-34 is expressed in the equatorial lens epithelium and that disruption of AND-34-mediated signaling in this cell type results in disruption of lens fiber organization. Shortly after birth, analysis of AND-34^−/−^ mice reveals cortical lens vacuolization and liquefaction followed several weeks later by rupture of the posterior lens capsule.

## Methods

### Lambda phage clones containing *AND-34*

A phage library representing the 129 mouse genome (Stratagene, La Jolla, CA) was screened for *AND-34* inserts using the entire cDNA sequence as a hybridization probe [2]. Positive clones were mapped for restriction sites and the maps compared to the known C57BL/6J genomic sequences (ENSEMBL mouse database) to further delineate exon locations. The number and location of exon borders was ascertained by comparison of the genomic sequences to the *AND-34* cDNA sequence [2]. Exons within the phage clones that were used in the knockout strategy were further sequenced to confirm exon/intron borders.

### Targeting vector construction

**Figure 1:**
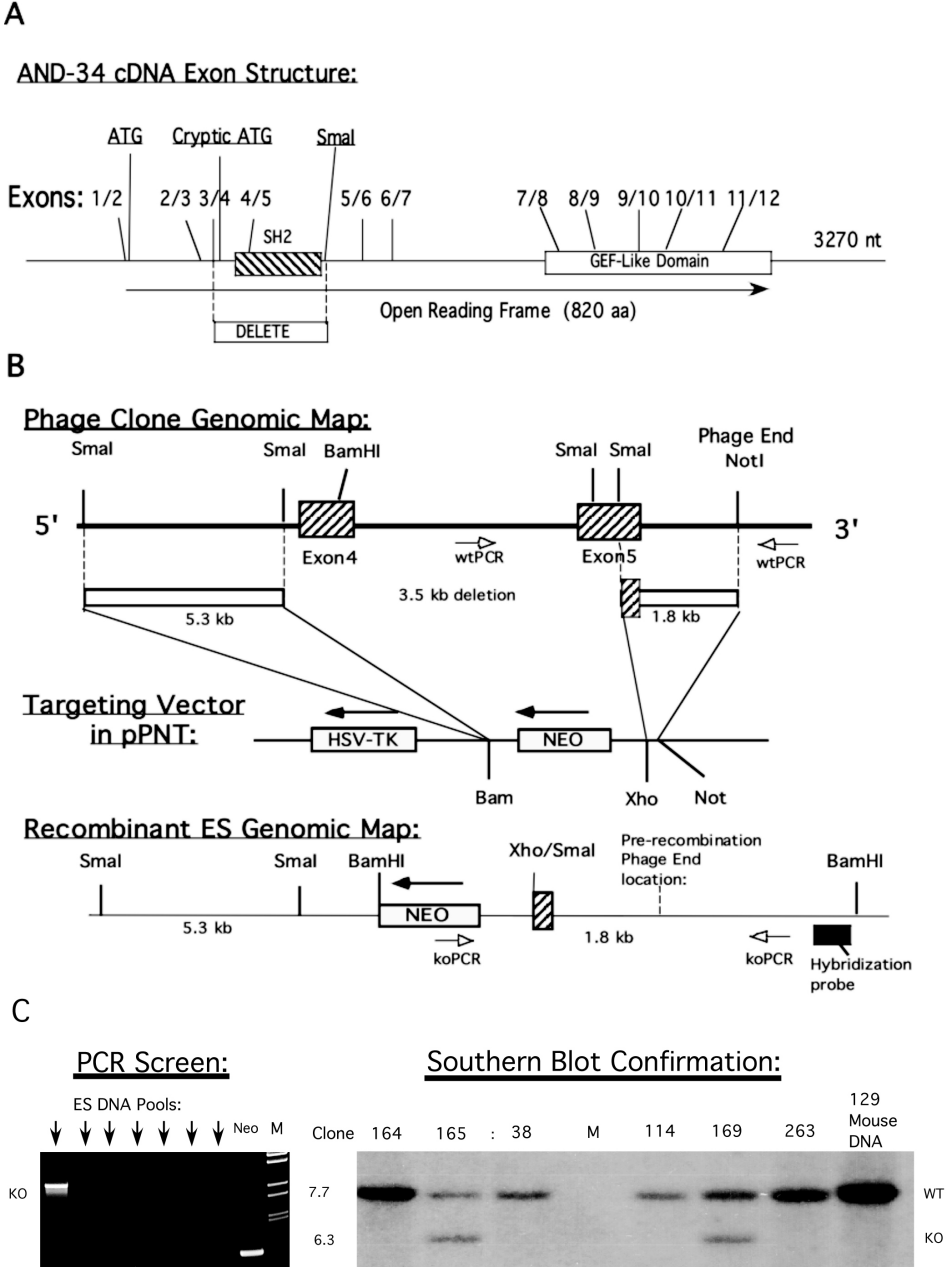
*AND-34* targeting. **A**: Exon structure of *AND-34* cDNA is shown. **B**: Production of the recombinant ES clone is demonstrated. A genomic phage clone containing exons 4 and 5 was mapped as shown. The area between the indicated SmaI sites (3.5 kb) was deleted, and the two surrounding “arms”, 5.3 kb of the 5′ sequence and 1.8 kb of the 3′ sequence, were cloned into the pPNT vector. Location of wild type and knockout PCR primers are shown as well as the hybridization probe used for Southern blotting. **C**: Screening for homologous recombination. DNA prepared from ES clones was purified, aliquots from four clones per lane were pooled, and PCR was performed to detect the recombined KO allele. The first lane shows a pool that contains an ES clone that has the KO allele (arrows indicate lanes of PCRs with clones that contain no AND-34^−/−^ allele). The control PCR used primers specific for *Neo*. Homologous recombination was confirmed by Southern blot analysis as shown. DNA from individual clones was cleaved with BamHI and analyzed with the indicated probe. Clones 165 and 169 display the recombinant allele. Wild type 129 mouse DNA serves as a control. M=marker lane.

Targeting vectors were created within plasmid pPNT [21] that contains Neo (positive selection) and Hsv-Tk (negative selection). An initial targeting vector (not shown) eliminated exons 1 and 2 (Figure 1A) with the strategy of deleting the start codon. Given that this initial approach resulted in a strain of mice that expressed a truncated form of *AND-34*, a subsequent alternate strategy was undertaken to eliminate exons 4 and 5 that contain the SH2 region of *AND-34* (Figure 1B). The 5.3 kb SmaI fragment 5′ of exon 4 was cloned into the BamHI site of pPNT using BamHI linkers. The 1.8 kb SmaI-NotI fragment containing a small portion (122 nt) of exon 5 (446 nt) was cloned into the XhoI-NotI portion of pPNT using a XhoI linker. These two “arms” encompassed a 3.1 kb segment bound by SmaI sites, which was omitted from the vector, thereby, eliminating the entire SH2 region.

### Homologous recombination in embryonic stem cells

ES line, TC1, kindly provided by Philip Leder (Harvard Medical School, Boston, MA) and derived from the 129S6/SvEvTac strain of mice, was used in these experiments. The conditions for electroporation, growth, and drug selection were essentially as described [22]. Briefly, the linearized (NotI) targeting vector (40 μg) was electroporated (250 V and 250 μF; Gene-Pulser; Bio-Rad, Hercules, CA) into TC1 cells (1×10^7^ cells) with subsequent selection for homologous recombination with 260 μg/ml G418 and 0.2 μM 1-(2-deoxy-2-fluoro–arabinofuranosyl)-5-iodouracil (FIAU; lowering to 0.1 μM after two days). The cells were cultured in Dulbecco’s modified Eagle’s medium (DMEM; Mediatech, Manassas, VA) supplemented with 15% ES-grade fetal bovine serum ((Sigma Aldrich, St. Louis, MO), 2% L-glutamine, 1% nonessential amino acids, 1% penicillin, and 1% streptomycin supplemented with 5×10^5^ U/500 ml Esgro-LIF (Millipore, Billerica, MA). Feeder cells were mitomycin-treated mouse embryonic fibroblasts from neomycin resistant mice. The medium was changed daily. Drug-resistant ES colonies were picked based on the lack of differentiation and size. Clones were expanded for freezing and DNA analysis. ES cell DNA was prepared using the DNeasy tissue kit (Qiagen, Valencia, CA).

### Polymerase chain reaction and Southern blot analysis

Platinum High-Fidelity Taq (Invitrogen, Carlsbad, CA) was used for genomic polymerase chain reaction (PCR) according to the manufacturer’s instructions. Only three primers were needed to assay for wild type and knockout alleles since one primer (KOIIWT3′) was used in both assays. The primers were 5PRGenNeoko 5′-TAC CGG TGG ATG AGG AAT GTG TGC GAG-3′, KOIIWT3PR 5′-GGA AAG GTA GAG GGT GAC TTG GAG G-3′, and KOIIWT5PR 5′-GTG TGG TAA TAC ATG GAG TGG AGA G-3′. Wild type (wt) was assayed with primers, KOIIWT5′ and KOIIWT3′, yielding a 2.4 kb fragment whereas knockout (KO) was assayed with 5′ GenNeoko and KOIIWT3′ yielding a 2.0 kb fragment. Southern blots also used DNA (10 μg) from either ES cells or tail digested with BamHI. DNA was transferred to GeneScreen Plus (PerkinElmer, Boston, MA) as suggested by the manufacturer and hybridized to ^32^P-labeled probe (Prime-a-Gene; Promega, Madison, WI).

### Implantation and generation of AND-34**^−/−^** mice

Cells from one recombinant ES clone were microinjected into C57BL/6J blastocysts. The injected blastocysts were implanted into oviducts of pseudopregnant foster mother Swiss Webster mice, resulting in six chimeric founder mice. All six founder chimeras were high-grade as indicated by the large percentage of agouti coat color. The three best founders were bred with wild-type C57BL/6J females to test for germline transmission of the targeted *AND-34* allele (Bcar3^tm1Lern^). F1 agouti offspring were found, indicating that the 129 ES cells populated the germ cells. Further, tail DNA was purified (DNeasy tissue kit; Qiagen) and screened by PCR for both wild type and knockout alleles. F1 agouti offspring with the KO allele were found indicating germline transmission. These mice were backcrossed for 10 generations to C57BL/6J mice to generate the congenic line, C57BL/6J-Bcar3^tm1Lern^. Further, founder chimeras were backcrossed to 129 female mice to directly obtain genetic homogeneity in 129 backgrounds. Animals were maintained at Boston University Medical Center (Boston, MA) in accordance with IACUC regulations.

### Tissue histopathology

Lung, liver, heart, kidney, brain, spleen, lymph node, thymus, and mammary gland from AND-34**^−/−^** and AND-34**^+/+^** mice were dissected and fixed in OmniFix (Xenetics Biomedical Inc., Tustin, CA). Fixed tissues were stained with hematoxylin and eosin and examined in the histopathology laboratory of Dr. Robert Cardiff (University of California Davis, Davis, CA). Immunophenotypic analysis of the thymus, lymph node, and spleen B and T cell populations was performed by standard techniques as previously described [1]. Eyes were removed and fixed with a modified phosphate-buffered glutaraldehyde-paraformaldehyde solution. They were then rinsed in buffer and processed routinely for paraffin or plastic embedding and sectioning as previously described [23].

### Western blots and immunoprecipitation

Non-eye tissues were lysed with a hand-held homogenizer (PowerGen 125; Fisher Scientific, Pittsburgh, PA) using a previously described lysis buffer (1% NP-40, 150 mM NaCl, 50 mM TrisCl (pH 7.4), 1 mM NaVO_4_, 1 mM EDTA, 1 mM EGTA, 5% glycerol, 25 mM glycerophosphate, and protease inhibitors) [9]. Lysates were incubated at 4 °C for 20 min with rotation. After centrifugation at 14,000 rpm (20,800x g) for 12 min, the supernatants were collected and processed, and western blots were performed as previously described [9]. For immunoprecipitations, 20 μl of protein G-Sepharose beads (GE Healthcare , Piscataway, NJ) and 2.6 μg anti-AND-34 antibody (see antisera section below) were added to cell lysates followed by end-over-end rotation for 3 h at 4 °C. Beads were pelleted by centrifugation, washed three times with lysis buffer, suspended in 40 μl of SDS-PAGE loading buffer, and heat denatured at 100 °C for 5 min. For eye tissues, eyes were dissected from AND-34**^−/−^**and AND-34**^+/+^**mice and placed in DMEM media on ice with no supplements. Under a dissecting scope, whole lenses were pushed out from a small incision. With fine forceps and scissors, the lens capsule was peeled away from center fiber cell material and placed separately in phosphate buffered saline (PBS; Mediatech Inc.) on ice. Both epithelial capsules and center fiber cells were lysed in the above lysis buffer with a small glass Dounce homogenizer (Fisher Scientific). Lysates were incubated at 4 °C for 30 min with rotation before processing as described above.

### Antisera

Two anti-AND-34 (NM_013867) and one anti-CHAT (NM_013781) antisera was prepared in rabbits with peptides cYEKQLKPFSKLLHEGREST (residues 680–699), KSPLAERRTDAYQDVSIc (residues 31–47), and cTALSHKLEPAIRSSEL (residues 687–702), respectively (Biosource International/QCB Immunology, Camarillo, CA). Due to a problem with antiserum stability, exon 2 of AND-34 knockout mice was examined using the cYEKQLKPFSKLLHEGREST antiserum while the exon 4/5 of AND-34 knockout mice was examined using the KSPLAERRTDAYQDVSIc antiserum (cysteine used for conjugation purposes denoted with a “c”). Polyclonal rabbit anti-Akt (9272) and anti-phospho-Akt Ser 473 (9271) were from Cell Signaling (Beverly, MA). Mouse anti-HA (MMS-101P) was from Covance (Princeton, NJ). Anti-p130Cas (sc-20029), anti-β-crystallin, and anti-fibronectin (sc-18825) antibodies were from Santa Cruz Biotechnology (Santa Cruz, CA). Polyclonal rabbit anti-tubulin was from Sigma (T3526). In some experiments, anti-p130Cas was from BD Biosciences (610271; San Jose, CA) since it also detects HEF1.

### In situ hybridization

Adult C57BL/6J mice (two months old) were anesthetized and perfused with 4% paraformaldehyde buffered with PBS. Tissues were then embedded in paraffin, sectioned (5 μm), and mounted on microscope slides by standard methods. A 826 nt BamHI fragment (nucleotides 1816–2642) from *AND-34* cDNA was subcloned into p11Z(-), which contains T7 and SP6 primer sequences. T7 RNA polymerase priming generated a ^35^S-labeled probe that detected antisense sequence whereas SP6 priming created a ^35^S-labeled probe that detected sense sequence such as those found in mRNA. The probes were synthesized with Riboprobe In Vitro Transcription System (Promega, Madison, WI) as per manufacturer’s instructions with endpoints generated at the borders of the inserts with EcoRI for SP6 and XbaI for T7. Riboprobes were resuspended in 20 μl of 1 M DTT and 180 μl of hybridization buffer (HB; 20mM Tris-HCl, pH 7.4, 5 mM EDTA, 10 mM Na_2_HPO_4_, 50% deionized formamide, 300 mM NaCl, 1X Denhardt’s solution, 10% dextran sulfate, and 0.5 mg/ml of total yeast RNA). Tissue sections were deparaffinized in xylene, rehydrated using a graded ethanol series (100%−30%) and rinsed in 150 mM NaCl and PBS, each for 5 min and post-fixed in 4% paraformaldehyde in PBS for 30 min. Slides were treated with proteinase K (20 μg/ml; Roche, Nutley, NJ) in 50 mM Tris-HCl, pH 7.2, containing 5 mM EDTA at room temperature for 7.5 min and fixed in 4% paraformaldehyde in PBS. Acetylation was performed for 10 min with 0.25% acetic anhydride in 100 mM triethanolamine, pH 8.0, and slides were rinsed sequentially in PBS and 150 mM NaCl, dehydrated through a graded ethanol series, and allowed to dry at least 2 h before hybridization. Hybridization was performed as previously described [24]. ^35^S-labeled probes were adjusted to 25,000 cpm/μl with HB before use. Probes were applied directly onto tissue sections, and the sections were covered with coverslips (22 mm×22 mm, 30 μl of probe/slip). Hybridization was performed at 52 °C for 16 h in a humid chamber. Slides were washed in 5X SSC containing 10 mM DTT at 50 °C for 30 min and subsequently at 65 °C for 20 min in 2X SSC containing 50% formamide and 10 mM DTT. Slides were rinsed twice at 37 °C for 10 min in STE (10 mM Tris-HCl, pH 7.5, 400 mM NaCl, and 5 mM EDTA). Sections were treated with RNase A (20 μg/ml) in STE at 37 °C for 45 min and washed with STE for 5 min, with 2X SSC for 15 min, and with 0.1X SSC for 15 min, each at 37 °C. Sections were dehydrated in an ethanol series (30%–95%) in 0.3 M ammonium acetate and finally in 100% ethanol. Slides were processed for standard light microscopic autoradiography using NTB-2 nuclear track emulsion (Kodak, Rochester, NY) and placed in a desiccation chamber for 8–10 weeks at 4 °C. The slides were developed in Kodak D-19 developer, stained with hematoxylin and eosin, and examined using a Leitz Dialux 20 light microscope (Leitz, Wetzlar, Germany).

## Results

### *AND-34* targeting

**Figure 2:**
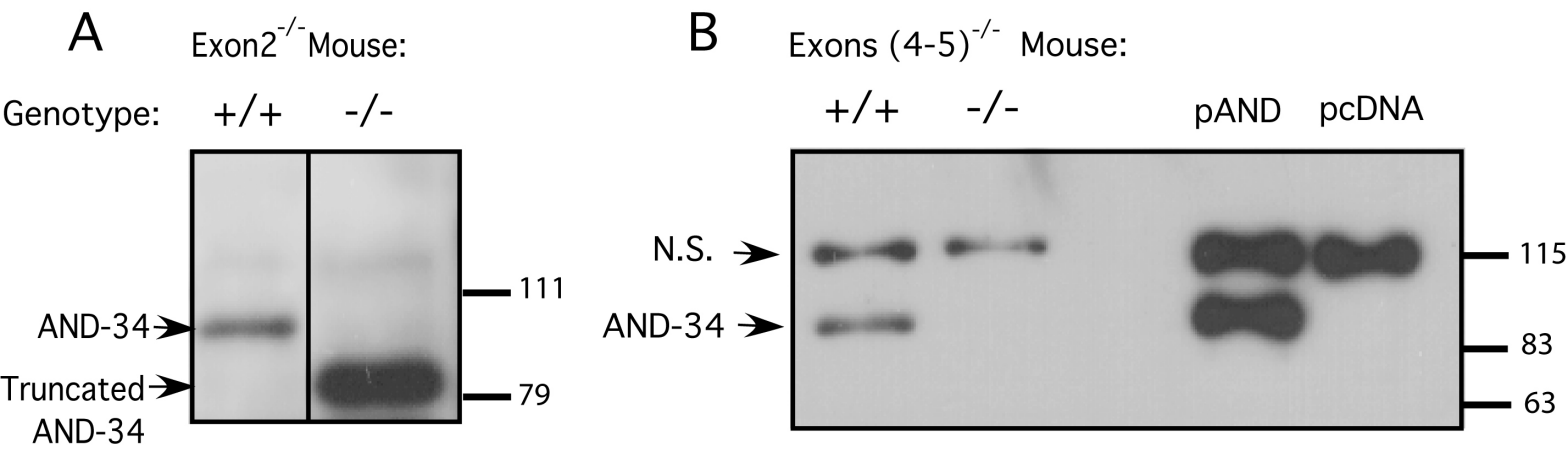
Exons 4 and 5 of AND-34^−/−^ mice lack AND-34 protein. Immunoblots of whole cell lysates from splenic lymphocytes from wild type (+/+) and knockout (−/−) mice were performed with two different polyclonal anti-AND-34 antisera. As positive and negative controls, lysates of 293T cells transfected with an AND-34 expression plasmid (pAND) or vector alone (pcDNA) were also included. **A**: AND-34^−/−^ mice in which exon 2 had been targeted were assessed with antiserum to peptide cYEKQLKPFSKLLHEGREST. The presence of residual truncated AND-34 protein product is noted in knockout splenic lymphocytes. **B**: AND-34^−/− ^mice in which exons 4 and 5 had been targeted were examined with antiserum to peptide KSPLAERRTDAYQDVSIc. The AND-34 protein is absent in knockout splenic lymphocytes. A non-specific band (N.S.) is detected with this antiserum.

To generate AND-34**^−/−^** mice by homologous recombination, we initially chose to remove exon 2 that contains the start codon of the *AND-34* cDNA [2]. A targeting construct in pPNT containing this deletion was homologously recombined with *AND-34* in ES cells, and mice lacking exon 2 of *AND-34* were successfully generated (Figure 1A). However, when analyzed for protein content in splenic lymphocytes, a truncated AND-34 protein was observed (Figure 2A). Analysis of the *AND-34* cDNA sequence revealed the presence of a cryptic start codon in exon 4 (Figure 1A). Although the truncated protein derived from the alternate start site lacks the first 120 amino acids, it contains all the known functional domains of AND-34 and the resultant knock-out mice showed no discernable phenotype.

Therefore, a second targeting strategy was used. In functional studies of AND-34 such as its ability to induce Rac activation, the SH2 region is required [19]. Elimination of exons 4 and most of 5 completely removes the SH2 region (Figure 1B). Alternate splicing of exon 3 with exon 6, 7, or 8 would also generate an out-of-frame sequence and premature termination codons, which preclude production of functional truncated protein products. A lambda phage clone from a 129 murine genomic library was isolated, which contained exons 4 and 5. The 3.5 kb area between two SmaI sites was eliminated and replaced by the *Neo* gene in vector pPNT (Figure 1B). Following transfection into the 129 ES line TC1 and selection in G418 and FIAU, two of the resulting 160 drug resistant clones (165 and 169) were identified as recombinants by a PCR screening strategy (Figure 1C). The recombinant genome was confirmed by Southern blot analysis (Figure 1C).

Clone 165 was chosen for microinjection into C57BL/6J blastocysts. The chimera progeny showed high penetrance of the 129 ES cells as judged by the agouti coat color. Breeding of these chimeras with C57BL/6J yielded F1 mice with agouti coat color indicated germline transmission. Tail genomic PCR confirmed the presence of the AND-34**^−/−^** allele (not shown). AND-34**^−/−^** founder mice were backcrossed to 129 mice to immediately establish genetically homogeneous mice or backcrossed to C57BL/6J mice for 10 generations. Since B cells from the spleen show high levels of *AND-34* expression, western blot analysis of splenic lymphocytes was performed to confirm loss of protein expression in AND-34**^−/−^** mice [4]. This analysis demonstrated that AND-34**^−/−^** splenic lymphocytes have indeed lost AND-34 protein expression (Figure 2B).

### AND-34 expression pattern in organs

**Figure 3:**
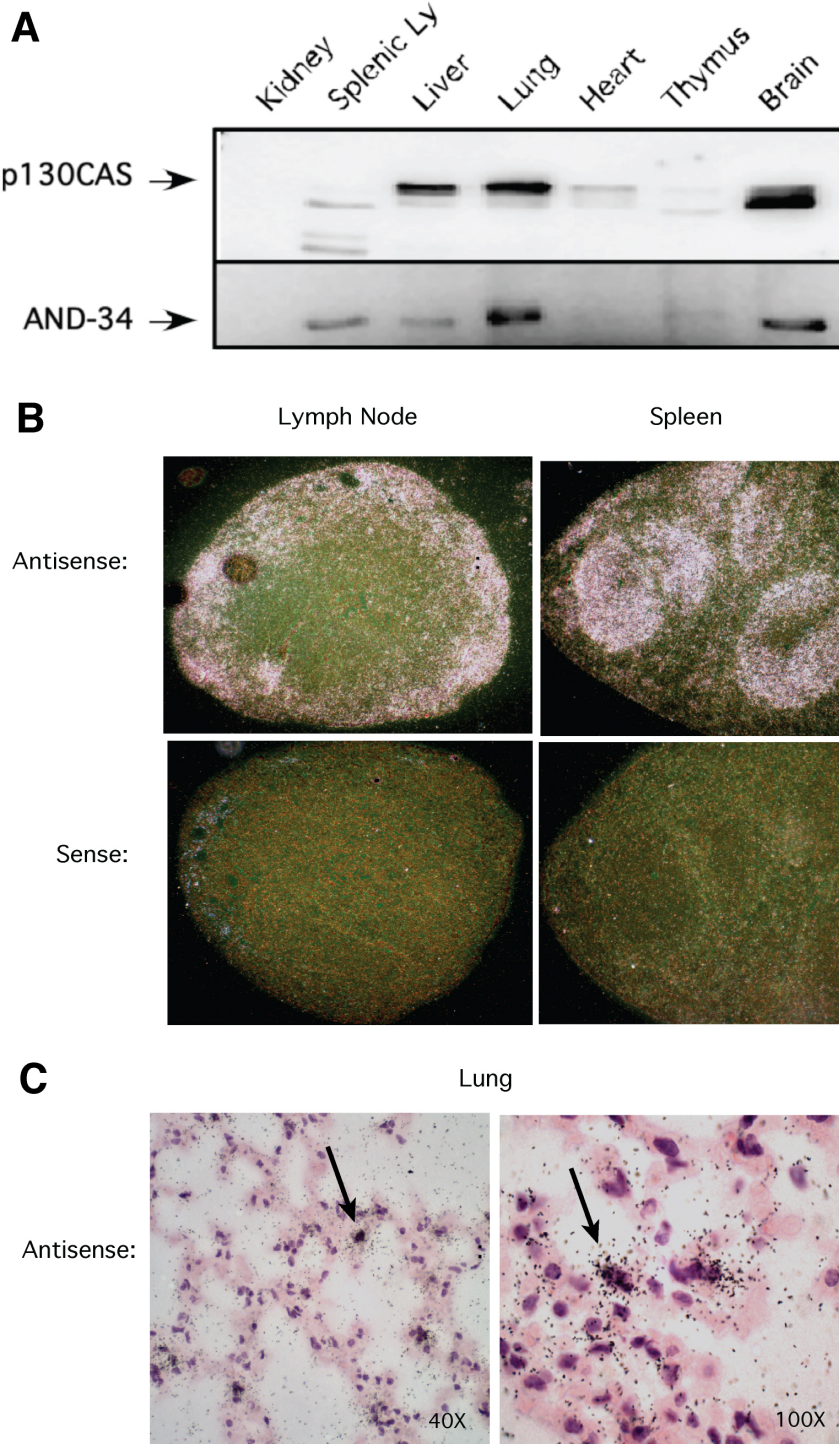
AND-34 expression in mouse tissues. **A**: Western blot analysis of wild type mouse tissues is shown. AND-34 was immunoprecipitated directly from lysates of mouse organs with polyclonal anti-AND-34 antibody whereas p130CAS was detected directly from whole cell lysates with a monoclonal anti-p130CAS antibody that is also able to detect HEF1. **B**: In situ hybridization for *AND-34* transcripts from lymph nodes and spleens of AND-34^+/+^ is displayed. The tissues were probed with sense or antisense hybridization probes as shown in dark-field microscopy. With dark-field illumination, the positive hybridization signal of the silver grains in the autoradiographic emulsion appears as white dots over a black background. **C**: In situ hybridization for *AND-34* transcripts in the lungs of AND-34^+/+^ mice is shown in bright-field microscopy. With bright-field illumination, the positive hybridization signal appears as black dots. Hybridization was detected in a subset of alveolar cells with the morphology of lung fibroblasts. Arrows indicate a strong hybridization signal.

Since the overall protein expression profile of AND-34 in various mouse organ systems has not been well characterized and since such a profile could suggest which tissue functions might be affected by loss of AND-34 expression, western blot analysis for AND-34 expression was performed using lysates from murine organs. Significant levels of AND-34 protein expression were seen in the spleen, lungs, brain, and liver (Figure 3A). However, AND-34 was difficult to detect in the thymus, heart, and kidney. The highest levels seen were in the lungs and brain. p130Cas, the focal adhesion adaptor protein and AND-34 binding partner, was also assayed. Expression of AND-34 and p130Cas was discordant as some tissues had detectable p130Cas but no AND-34 (heart) while the splenic lymphocyte sample had detectable AND-34 but relatively little p130Cas (Figure 3A). In splenic lymphocytes, a 105 kDa immunoreactive species was also detected, which corresponds to the p130Cas homolog, HEF1 [4]. As p130Cas was not effectively immunoprecipitated from kidney whole cell lysates, levels of AND-34 were not assessable in the kidney from this analysis.

To complement the protein expression assays, the presence of *AND-34* RNA was assayed by in situ hybridization with ^35^S-labeled sense and antisense riboprobes. In prior studies, western blot analyses have demonstrated that AND-34 is expressed in murine splenic B lymphocytes but not T lymphocytes. Consistent with such work, the in situ hybridization studies demonstrate that in the spleen, AND-34 expression is limited to the periphery of the white pulp and appears to be absent from the central T cell-rich periarteriolar lymphoid sheath (PALS) region (Figure 3B). In lymph nodes, the preponderance of *AND-34* transcript expression occurs in the cortical region, again a region known to be enriched for B lineage lymphocytes. The pattern of *AND-34* expression was also investigated by in situ hybridization in the lungs. *AND-34* expression was detected in a subset of alveolar cells, possibly the lung fibroblasts, as the cells frequently had elongated nuclei characteristic of this cell type (Figure 3C).

### AND-34**^−/−^** mice have few phenotypic abnormalities

AND-34**^−/−^** mice were initially observed to be healthy and without overt signs of an altered phenotype. No size or behavioral differences were noted. Litter sizes were unchanged with no skewed male/female ratios seen. AND-34**^+/−^** × AND-34**^+/−^** mating showed typical Mendelian genetics with AND-34**^−/−^** mice comprising about a quarter of such litters. Further, there were no overt longevity differences. No obvious change in the incidence of spontaneous tumors was noted in AND-34**^−/−^** mice.

Tissue samples from both AND-34^+/+^ and AND-34**^−/−^** adult mice including the lungs, liver, heart, kidney, brain, spleen, lymph nodes, thymus, and mammary glands were removed for histopathological analysis. No abnormalities in organ systems were detected. As murine splenic B cells show high levels of AND-34 protein expression, thymic (CD4, CD8, and B220), splenic (IgM, IgD, B220, CD4, CD8, CD21, CD9, CD1d, CD23, and CD19), and peritoneal (B220, Mac1, and CD5) lymphoid populations were assessed by immunophenotyping experiments [4]. No consistent abnormalities in the proportions of B and T cell populations in these organs were observed in AND-34**^−/−^** mice (data not shown).

### AND-34**^−/−^** mice undergo postnatal lens rupture

**Figure 4:**
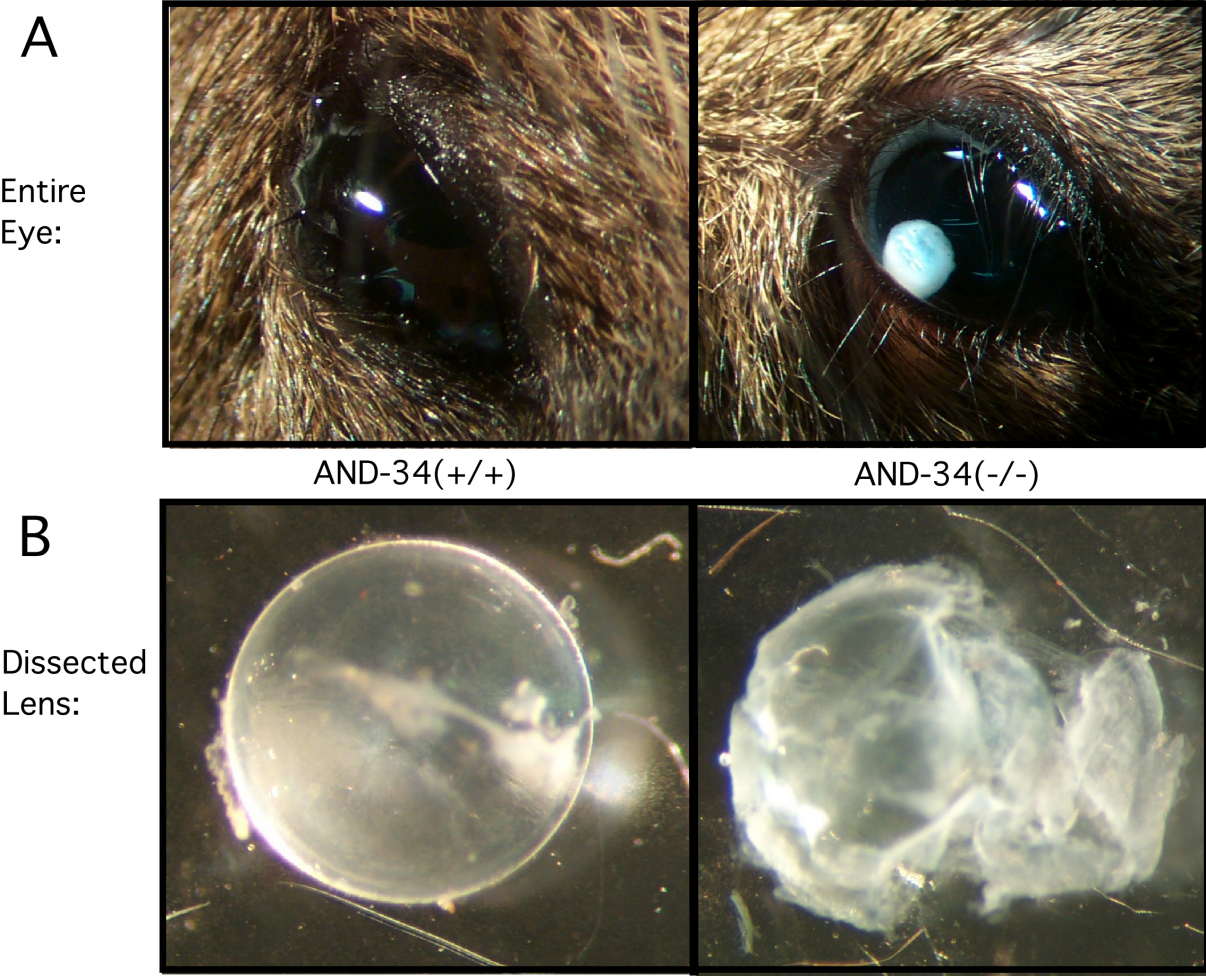
Ocular phenotype in AND-34^−/−^ mice. **A**: The lens cortex was observed in the anterior chamber of the eye in a subset of AND-34^−/−^ mice (four-month-old mouse shown on right). Comparable abnormalities were never observed in AND-34^-/+^ or AND-34^+/+^ mouse eyes (left). **B**: Dissected whole lens from a three-month-old AND-34^−/−^ 129 strain mouse was compared with a three-month-old AND-34^+/+^ lens as viewed under a dissecting microscope. The AND-34^+/+^ lens was a uniform translucent, smooth, spherical structure whereas the lens from the AND-34^−/−^ mouse eye was a partially opaque, irregular structure that had clearly lost a portion of its internal contents.

During general maintenance of the mouse colony, it was noted that some mice had small white circular lesions in the anterior chamber that were shown to be derived from the lens cortex (Figure 4A). Such eye abnormalities were observed in AND-34**^−/−^** 129 strain mice but not in their heterozygous littermates nor in mice with a C57BL/6J genetic background. In the 129 strain background, eye abnormalities were present in the progeny of two different founder mice. This anterior chamber phenotype was usually unilateral but occasionally bilateral. However, not all 129 AND-34**^−/−^** mice displayed this phenotype. In one survey, 15 out of 51 AND-34**^−/−^** mice had anterior chamber lens cortex material. Furthermore, the eye findings were not seen in young mice (less than three weeks old), suggesting an age-dependent process was responsible for this pathology. Dissection of eyes from adult AND-34**^−/−^** mice revealed that unlike the round, translucent lenses of their heterozygous littermates, the lenses from knockout animals were partially opaque, irregular structures that appeared to have lost a portion of their internal contents (Figure 4B). Unlike the anterior chamber lens cortex, this abnormal lens phenotype was observed in all adult AND-34**^−/−^** mice and was further present in mice of both 129 and C57BL/6J genetic background.

**Figure 5:**
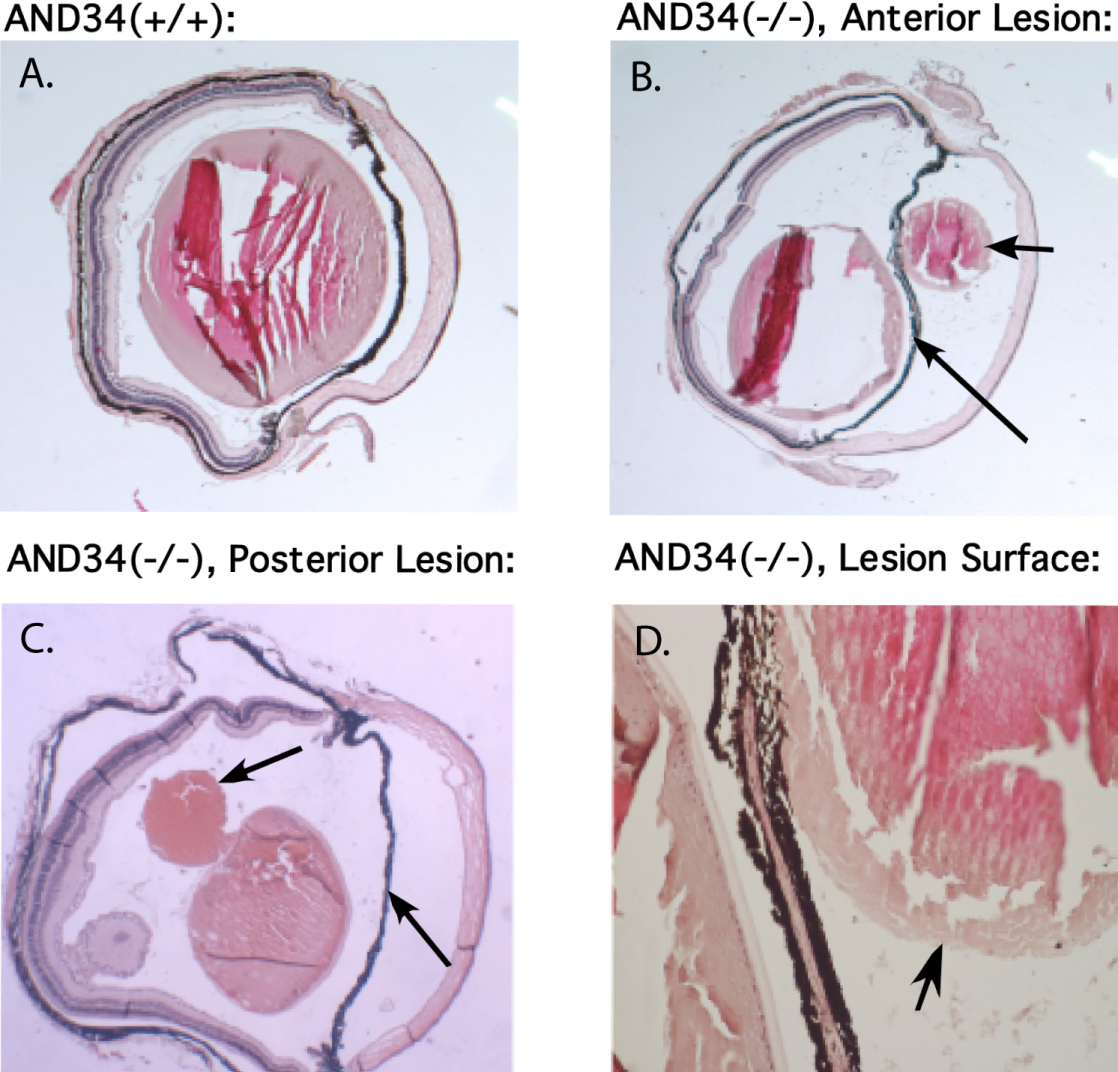
Paraffin-embedded histopathology of AND-34^−/−^ mouse eyes. Paraffin tissue sections of four-month-old AND-34^−/−^ eyes are compared with a four-month-old AND-34^+/+^ eye. Panel **B** shows a fragment of anterior chamber lens cortex (short arrow) in front of the iris (long arrow). Panel **C** shows a fragment of lens cortex (short arrow) behind the iris (long arrow). Panel **D** shows that the fragment of lens cortex lacks both epithelial cells and a lens capsule. The lens capsule is visible in the lens itself on the left side of this panel. In panels **A** and **B**, the loss of lens fiber material from the lens is due to artifactual shearing during the cutting of the paraffin section.

**Figure 6:**
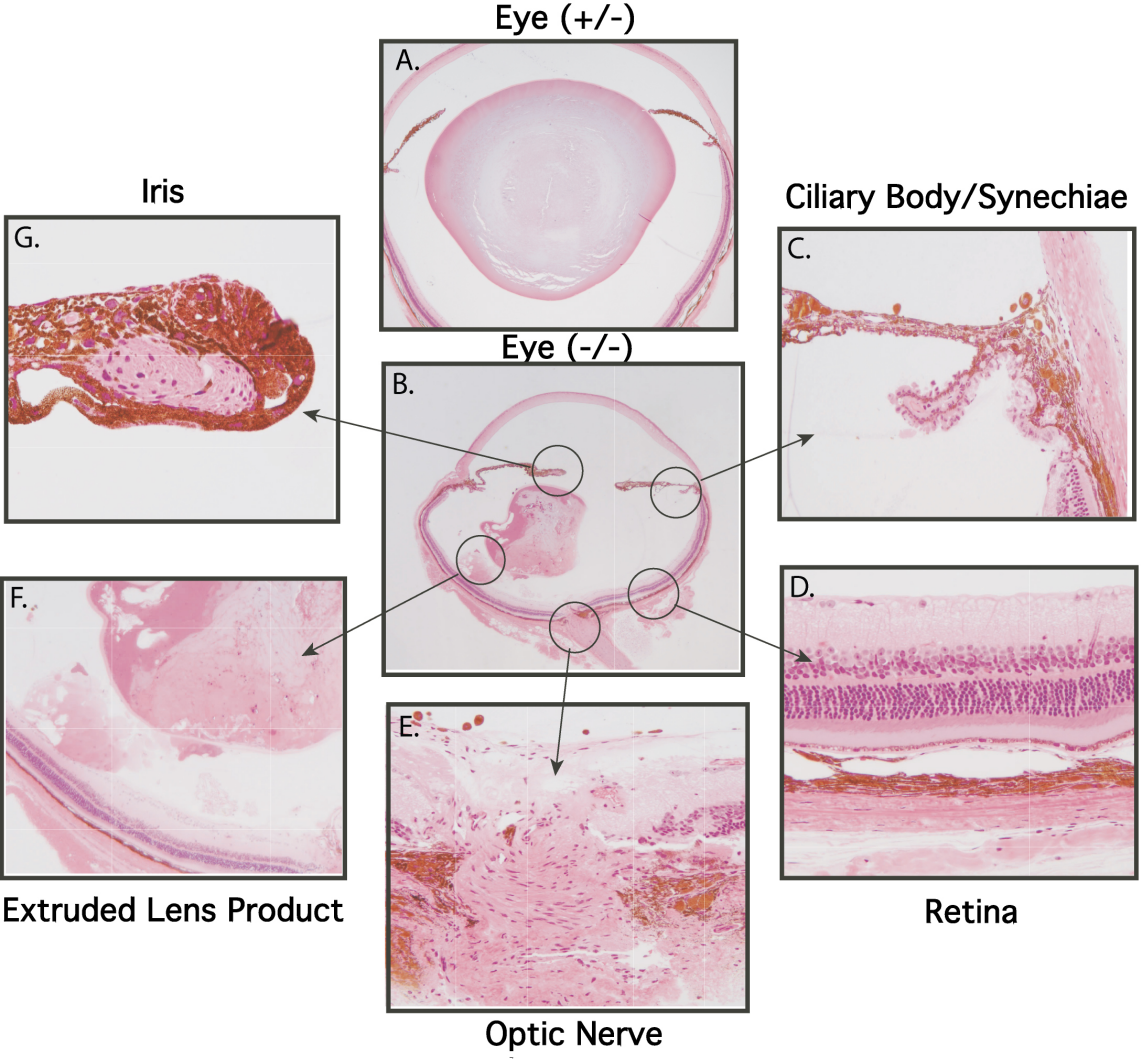
Plastic-embedded histopathology of AND-34^−/−^ mouse eyes. Tissue sections from three month-old mature heterozygous AND-34^+/−^ and homozygous knockout AND-34^−/−^ eyes are shown in Panels **A** and **B**, respectively. Abnormalities in the indicated regions of the AND-34^−/−^ eye are described in the text.

Initial histologic studies on paraffin-embedded AND-34**^−/−^** eyes showed that the anterior chamber phenotype was due to cortical lens fragments floating in the aqueous humor (Figure 5B). Extruded cortical lens material was also seen posterior to the iris (Figure 5C). Lens capsule and lens epithelial cells were absent from the anterior chamber cortical lens fragments (Figure 5D). No extruded lens cortex or cataracts were detected in AND-34^+/−^ or AND-34^+/+^ mice (Figure 5A and Figure 6A).

A subsequent analysis of plastic embedded eyes from two- to six-month-old adult AND-34**^−/−^** mice suggested that lens cortex had extruded through a ruptured lens capsule. In all adult AND-34**^−/−^** 129 strain mice examined, there was evidence of such lens rupture. In the majority of cases, lens cortex material was detected only posterior to the iris, thus explaining the puzzling lack of correlation between genotype and the anterior chamber phenotype. In addition to the lens rupture, the remaining lens showed a dense cataract with cortical liquefaction (Figure 6F). Other phenotypic alterations observed in adult AND-34**^−/−^** eyes included abnormally deep anterior chambers and anterior synechiaes covering the trabecular meshwork (Figure 6C; a likely secondary glaucoma), ectropion uveae (Figure 6G; central iris pigment epithelium migrating to the anterior surface of the iris), mild to moderate retinal ganglion cell loss (Figure 6D), and a small, pigmented pre-retinal membrane overlying the optic nerve (Figure 6E).

**Figure 7:**
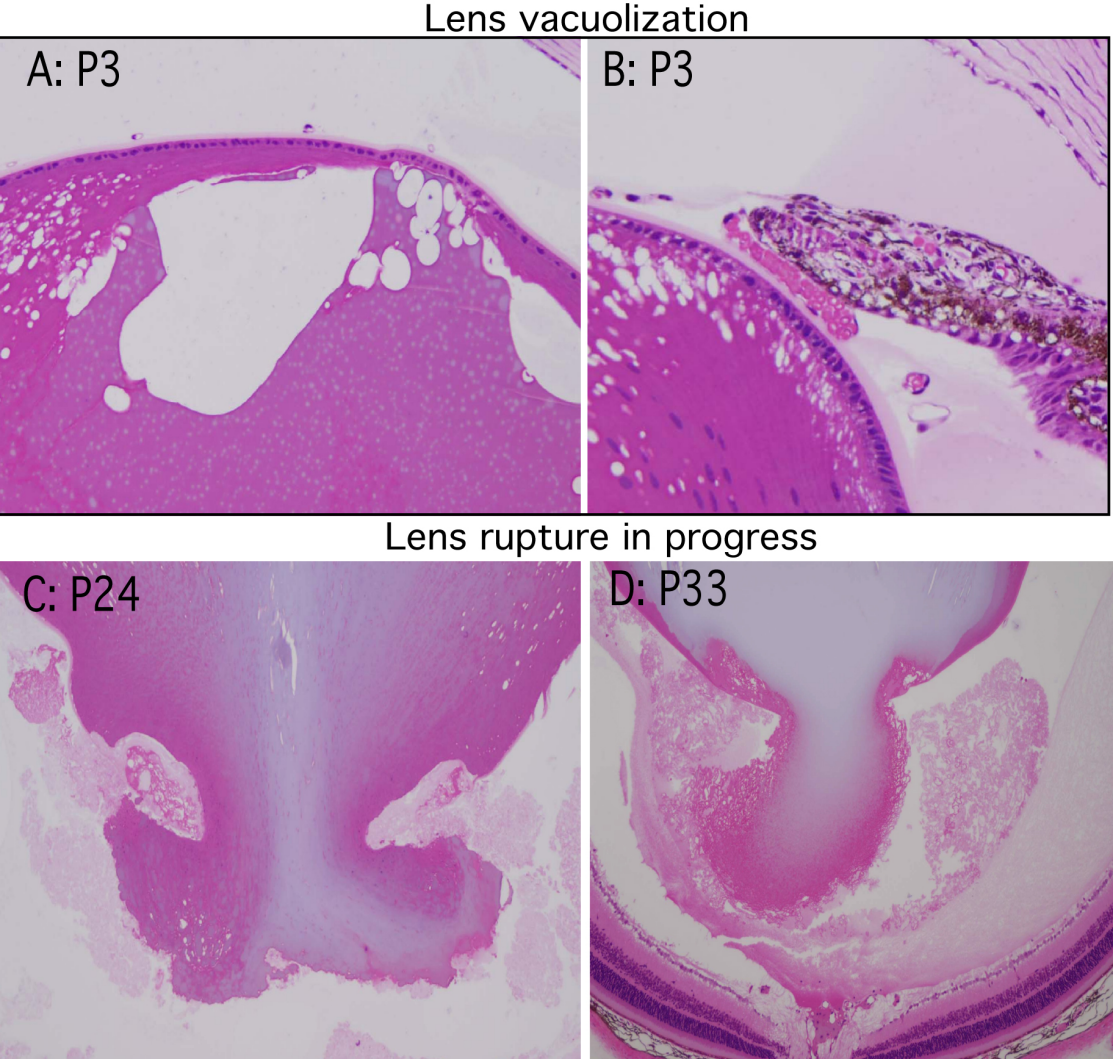
Lens rupture in AND-34^−/−^ mice occurs postnatally following initial development of lens vacuolization. AND-34^−/−^ eyes from 3-, 24-, and 33-day-old AND-34^−/−^ mice (P3, P24, and P33) were fixed, plastic embedded, and sectioned. Vacuolization of the cortical lens fibers but no examples of lens rupture were identified in the eyes of three-day-old mice (upper panels). Lens rupture with extrusion of cortical fiber material was first detected in 24- and 33-day old mice (lower panels).

Since the visible anterior chamber phenotype was not seen in mice younger than three weeks of age, we examined eyes from younger mice to follow the development of the adult phenotype. Mice three days old (P3) showed striking anterior lens vacuolization and in some cases liquefaction of lens cortical fibers (Figure 7A). Of note, in P3 mice, there were no obvious lens capsule defects and no lens extrusion. These findings confirmed that the lens defects occurred postnatally. However, in P24 mice, all of which showed extensive lens cortex vacuolation, one eye among three P24 mice examined demonstrated early lens extrusion (Figure 7C). By P33, all eyes showed at least equatorial cortical vacuolation and frequent examples of lens undergoing extrusion of cortical material (Figure 7D).

### *AND-34* and *CHAT* expression in murine lens epithelium

**Figure 8:**
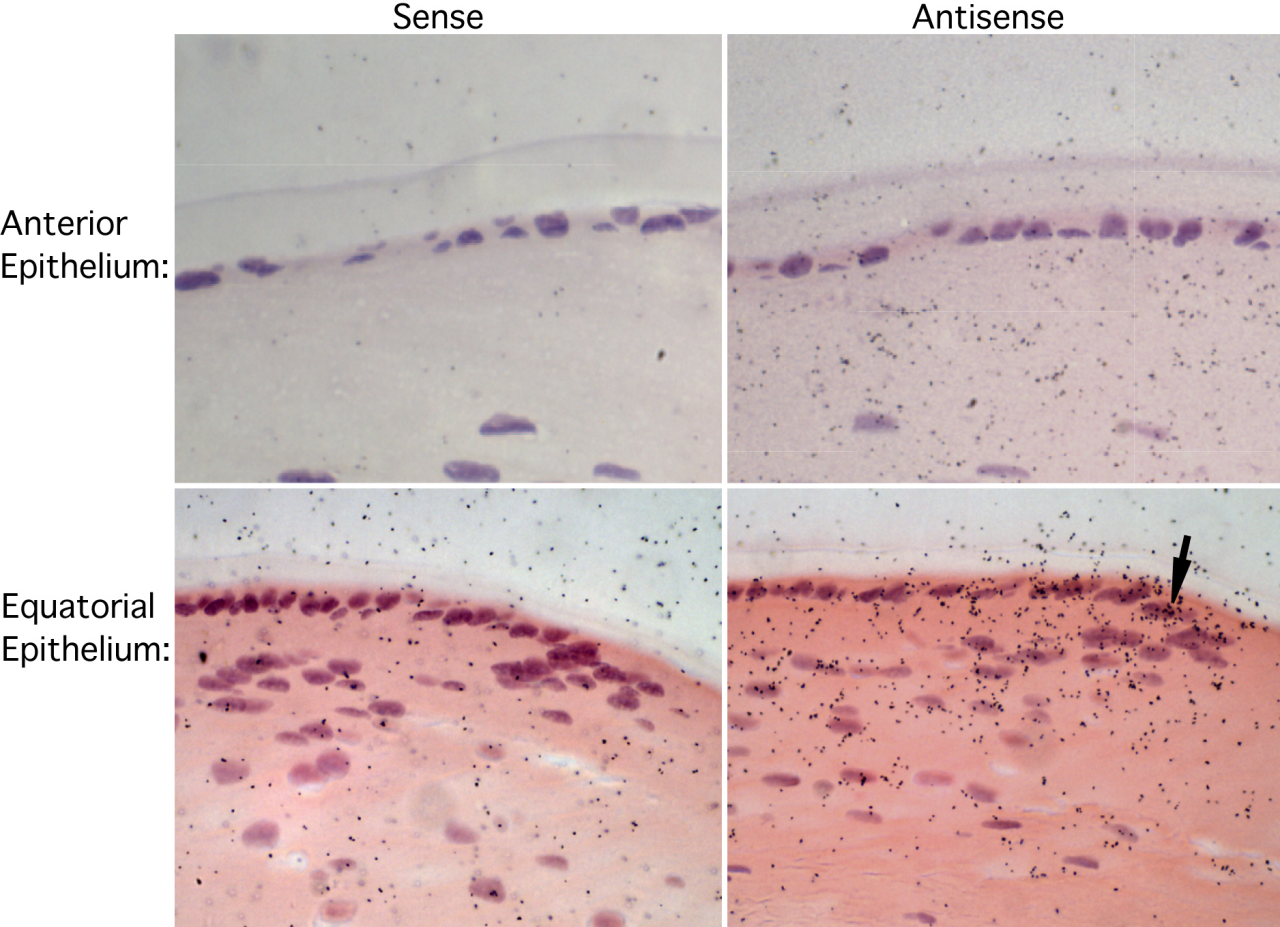
Expression of *AND-34* transcript in lens tissue. Eyes from AND-34^+/+^ mice (two months old) were fixed and probed with sense or antisense hybridization probes for *AND-34* transcripts. Expression of *AND-34* was detected by the antisense probe in the lens epithelial cells at the lens equator (lower panels) but was not reproducibly observed in the anterior epithelial cells (upper panels).

To characterize the role of AND-34 in the aberrant lens phenotype, lens tissue was examined for the presence of *AND-34* transcript by in situ hybridization. *AND-34* transcript expression was detected in lens epithelial cells, predominantly at the equatorial region where such epithelial cells are known to proliferate, migrate centrally, and differentiate into cortical lens fiber cells (Figure 8). In contrast to the lens epithelium, only the earliest nucleated cortical fiber cells demonstrated hybridization with the *AND-34* antisense riboprobe. The lack of *AND-34* transcript in the central lens fiber cells is not surprising as deep cortical lens fiber cells coincidentally lose both their nuclei and endoplasmic reticulum and therefore cannot transcribe RNA [25].

**Figure 9:**
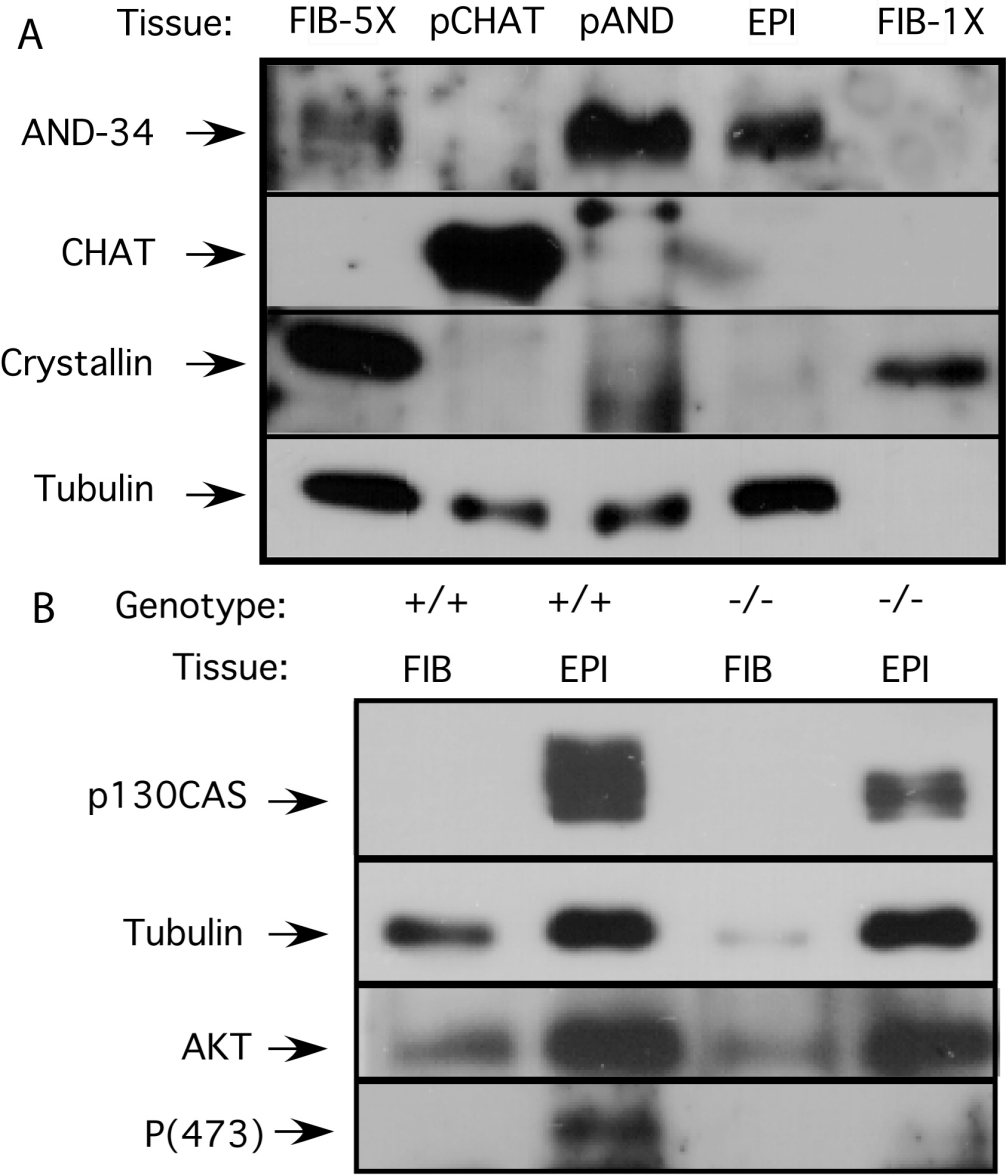
*AND-34* expression regulates p130Cas and Akt signaling in lens epithelial cells. Lenses were removed from the eyes of AND-34^−/−^ and AND-34^+/+^ mice (four months old), and the capsular epithelial layer (EPI) was separated from the lens fibers (FIB) by dissection. **A**: The expression of AND-34/NSP2 and CHAT/NSP3 were examined in the lens tissues of AND-34^+/+^ mice by immunoblot. Lysates from HEK293T cells that were transfected with AND-34 (pAND) or CHAT (pCHAT) expression constructs were used as controls. Expression of crystallin, a component of lens fibers, and tubulin were also examined. To detect low level expression of AND-34 in lens fibers, fivefold more of the lens fiber sample was loaded in the first lane (Fib-5X) than in the fifth lane (Fib-1X). **B**: The phosphorylation status of p130Cas and AKT Ser 473 was examined in AND-34^−/−^ and AND-34^+/+^ lens epithelial and lens fiber samples by western blot analysis. Phosphorylated p130Cas is characterized by reduced PAGE migration. Total levels of AKT expression were also assessed as a control. Similar results were obtained in three experiments.

*AND-34* is a member of a gene family of which there are two members in mice, *AND-34/NSP2* and *CHAT/NSP3*. To determine whether *CHAT* expression could serve a redundant biological role in the murine lens, we assessed AND-34 and CHAT protein expression in the lens epithelium and lens fiber cells from wild type mice. AND-34 was readily detected in western blots of lens epithelial cell tissue but was not detected when comparable amounts of lens fiber cell protein was examined (Figure 9A). As expected, β-crystallin was readily detected in lens fiber samples but not in the lens epithelial samples. Given that lens fiber cells but not lens epithelial cells contain an abundant amount of crystallin (see Figure 9A), it is not easy to compare the expression of other less abundant proteins between these two cell types by western blot analysis. Upon loading fivefold more lens fiber samples, AND-34 expression was detected (Figure 9A, lane 1). Neither lens epithelial nor fiber cells showed detectible CHAT expression.

### p130Cas and Akt S473 phosphorylation is reduced in the AND-34**^−/−^** lens epithelium

AND-34/BCAR3 induces serine phosphorylation of p130Cas in human breast cancer epithelial cells in an adhesion-dependent manner (unpublished observation). Such serine phosphorylation results in reduced p130Cas PAGE migration that is easily detected on western blot analysis. To determine whether expression of AND-34 might also regulate p130Cas phosphorylation in murine lens epithelium, we examined p130Cas in lens fiber and lens epithelial preparations from AND-34**^−/−^** and wild type mice. A significant portion of p130Cas in wild type lens epithelial cells ran as a more slowly migrating species, consistent with a phosphorylated form of p130Cas (Figure 9B). In contrast, this more slowly migrating form of p130Cas was absent in the lens epithelial cells from AND-34**^−/−^** mice.

As PI3K signaling have been implicated in both lens cell differentiation and AND-34 mediated Rac activation, we next sought to determine whether signaling by this pathway was altered in the lens epithelium from AND-34**^−/−^** mice [26]. PI3K activation results in phosphorylation of the serine/threonine kinase, Akt, at Thr308 by 3-phosphoinositide dependent protein kinase-1 (PDK-1) and at Ser473 by the TORC2 (target of rapamycin complex 2) complex [27,28]. Akt Ser473 phosphorylation was readily detectable in wild type lens epithelial cells but was markedly reduced in lens epithelial cell preparations from AND-34**^−/−^** mice (Figure 9B). Total levels of AKT protein were comparable in wild type and knockout lens epithelial cells, and AKT was detected at only low levels in lens fiber cells. These results demonstrate that loss of BCAR3 expression results in a reduction in both p130Cas and Akt Ser473 phosphorylation in murine lens epithelium.

## Discussion

We have demonstrated that AND-34**^−/−^** mice develop lens rupture approximately one month after birth while AND-34**^+/−^** mice have histologically normal lenses. Despite widespread expression of *AND-34* in the brain, liver, lungs, spleen, and thymus, the phenotype we have observed following the loss of *AND-34* expression is thus far restricted to the ocular lens. The obvious questions that this phenotype raises are why the lens rupture occurs and what accounts for the specificity of the tissue affected.

The characteristics of the lens rupture we observe in AND-34**^−/−^** mice are different from those of most previously reported forms of molecularly characterized lens defects. Among the four major forms of mouse cataracts, lens extrusion cataracts are the least common [29]. While several spontaneous murine lens extrusion cataract mutations have been described, the majority have not been characterized as to the specific gene affected. Further, among those in which the genetic locus has been identified, the degree of lens rupture has not typically been as severe as that observed in AND-34**^−/−^** mice [30-33]. A strain of mice with a spontaneously occurring recessive “lens rupture” (lr) mutation were reported in 1950 and has some characteristics similar to AND-34^−/−^ mice [30]. This albino strain of mice had posterior rupture of the entire nucleus of the lens with 100% phenotypic penetrance at three months. In striking similarity to AND-34**^−/−^** mice, either the displaced nucleus or the remaining soft degenerate lens passed into the anterior chamber in 18% of cases. In a second strain of mice reported in 1963, a radiation-induced “ectopic” (ec) lens rupture mutation resulted [31]. Unfortunately, the genetic basis for the lr and ec phenotypes was never established, and both strains of mice are now extinct. Secreted protein rich in cysteine (SPARC) is a matricellular protein whose elimination results in lens opacity at one month of age and cataract formation and lens capsule rupture at seven to eight months of age. However, overall, the severity of the SPARC^−/−^ phenotype appears considerably less than that observed in AND-34**^−/−^** mice [34]. Loss of Abi2, a member of the Abl-interactor family of adaptor proteins, results in abnormal lens fiber orientation during development, failure of anterior and posterior lens suture formation, and posterior lens rupture at postnatal day 1 [35]. We have not observed major abnormalities in lens fiber orientation or suture formation in AND-34**^−/−^** mice, suggesting that the rupture may not occur as a result of improper alignment of developing lens fibers.

Since the first lens abnormality observed is cortical lens fiber vacuolization followed by lens fiber liquefaction, we hypothesize that disruption of normal focal adhesion complex dynamics in AND-34**^−/−^** lens epithelial cells and their differentiated progeny result in defective adhesion between lens fibers. Cortical lens fiber vacuolization and liquefaction is eventually followed by lens capsule rupture and extrusion of cortical lens material into the posterior chamber of the eye. As is the case with most prior reports, lens rupture in AND-34**^−/−^** mice occurs posteriorly where the capsule is weakest. The lens capsule is one of the thickest basement membranes in the body and is composed of collagen, laminin, fibronectin, entactin-1, and sulfated proteoglycans [36]. The capsular matrix is produced and remodeled throughout life by lens epithelial cells anteriorly and by the basal ends of elongating fibers posteriorly [37]. As overexpression of AND-34 augments fibronectin deposition in breast cancer epithelial cell lines, we also hypothesize that loss of AND-34 in lens epithelial cells alters the composition and strength of the lens capsule [9]. Of note, since other animal models of cataract characterized by fiber breakdown do not necessarily undergo capsular rupture, it would appear that the altered synthesis of the lens capsule is likely to play a pivotal role in the lens rupture observed in AND-34**^−/−^** mice. As lens fibers elongate, they maintain an apical cell-cell interaction with lens epithelial cells and a posterior cell-extracellular matrix (ECM) interaction with the lens capsule. Given that we observe an extrusion of ruptured cortical material posteriorly, loss of AND-34 may dysregulate lens fiber interaction with and maintenance of the posterior lens capsule.

Our observation that loss of *AND-34* expression leads to the rupture of the adult lens is unexpected and suggests that interaction of AND-34 with other components of the focal adhesion complex plays a non-redundant role in maintaining the integrity of the lens. Focal adhesion kinase (FAK) transmits signals from integrins to the p130Cas complex by binding to the p130Cas SH3 domain. FAK expression in the adult lens is restricted to those areas where lens epithelial cells exit the cell cycle and initiate differentiation (posterior germinative zone and transitional zone, respectively) [38]. FAK is also present in the basal membrane complex of lens fiber cells where they attach to the lens capsule [39]. FAK in turn regulates Src-mediated regulation of p130Cas signaling. Src family kinases bind by their SH2 domains to the FAK Y397 autophosphorylation site, facilitating Src kinase activation and subsequent Src-mediated phosphorylation of multiple tyrosine motifs in the substrate domain of p130Cas [40]. Similar to the studies noted above with FAK, activated Src (phospho-Y527) is selectively observed in equatorial lens epithelia [41]. Our studies suggest that loss of AND-34 leads to lens rupture as a result of altered p130Cas complex signaling in the same equatorial lens epithelial population identified as the site of lens-associated FAK and Src signaling. Western blot analysis confirms *AND-34* expression in lens epithelial cells and to a lesser extent in lens fiber cells when normalized for total protein loaded. In situ hybridization demonstrates that within the lens epithelium, the predominant site of *AND-34* expression is at the lens equator. Altered PAGE mobility of p130Cas in preparations derived from AND-34**^−/−^** mice is consistent with reduced p130Cas phosphorylation. However, while most studies of p130Cas have focused on Src-mediated tyrosine phosphorylation of the p130Cas substrate domain, our own recent studies suggest that AND-34 regulates p130Cas serine phosphorylation (unpublished observation). Given limitations in the amount of protein we could obtain from primary AND-34**^−/−^** lens epithelia, studies with cultured lens epithelial cells from wild type and AND-34**^−/−^** mice will be required to confirm whether loss of AND-34 does in fact reduce lens epithelial p130Cas serine phosphorylation as well as the functional implications of such reduced phosphorylation. As the absence of *AND-34* transcript expression in lens fibers is an expected consequence of the general loss of transcripts from such cells and as we detect AND-34 protein expression in lens fibers by western blot analysis, it remains possible that loss of *AND-34* expression has an important impact on lens fiber function as well as lens epithelial cell function.

AND-34 induced Rac activation in breast cancer epithelial cell lines is dependent upon PI3K activation, most likely as a result of activation of pleckstrin homology domain-containing Rac GEFs. The same AND-34 mediated PI3K activation is associated with Akt signaling as judged by Akt Ser 473 phosphorylation [19]. Both PI3K itself and PI3K dependent Rac activation are known to play important roles in lens epithelial differentiation and survival [26]. Rac activation antagonizes Rho, resulting in the disassembly of actin stress fibers, a critical event in lens epithelial cell differentiation [42]. Among PI3K effector proteins, Akt is required for IGF-1 (insulin-like growth factor 1) mediated protection of lens epithelial cells from apoptotic stimuli [43]. PI3K signaling and Akt has also been implicated in differentiation of lens epithelial cells into secondary fiber cells in response to vitreal factors [44,45]. To examine whether an AND-34 dependent PI3K signaling pathway might be active in murine lens epithelia, we examined levels of Akt Ser 473 phosphorylation in lens epithelial lysates from wild type and AND-34**^−/−^** mice. Akt Ser473 phosphorylation was markedly diminished in lysates derived from AND-34 null mice despite conservation of total levels of Akt. These results support the hypothesis that loss of AND-34 disrupts a PI3K and Rac-mediated signaling pathway required for appropriate differentiation of lens epithelial cells. Further studies of AND-34 dependent PI3K and Rac signaling in cultured lens cells should help to elucidate the relationship of such signaling to lens rupture in AND-34**^−/−^** mice.
